# Non-attendance in Diabetes and Endocrinology Clinics: A Retrospective Observational Study of Inequalities in Outpatient Access

**DOI:** 10.7759/cureus.115804

**Published:** 2026-09-04

**Authors:** Dhaksha Seyone, Leanne Cheng, Sanjeev Mehta, Andrew Solomon

**Affiliations:** 1 Medicine, Imperial College London, London, GBR; 2 Diabetes and Endocrinology, Ealing Hospital, London North West University Healthcare NHS Trust, London, GBR; 3 Diabetes and Endocrinology, Northwick Park Hospital, London North West University Healthcare NHS Trust, London, GBR

**Keywords:** diabetes and endocrinology, health service utilisation, outpatient care, outpatient non-attendance, reducing missed appointments

## Abstract

This retrospective observational study investigated demographic and service-level factors associated with non-attendance in diabetes and endocrinology outpatient clinics. Data from 159 outpatient appointments were analysed. In unadjusted univariate analysis, attendance varied significantly by ethnicity (Fisher’s exact test, p = 0.002), with higher odds of non-attendance among patients identifying as Black [odds ratio (OR) 4.31, 95% confidence interval (CI) 1.28 - 14.46] and Other (OR 5.72, 95% CI 2.28 - 14.3) compared with White patients. Given the small subgroup sizes, wide confidence intervals, and absence of adjustment for socioeconomic and other confounders, these estimates should be interpreted as hypothesis-generating rather than evidence of an independent ethnic effect.

Morning appointments were associated with higher odds of non-attendance in a univariate logistic regression analysis (OR 1.92, 95% CI 0.81 - 4.50), though this did not reach statistical significance. These findings indicate demographic variation in outpatient attendance that warrants further adjusted investigation, while the role of appointment timing remains inconclusive.

## Introduction

In 2023-2024, 20.8 million outpatient appointments in England were not attended [1], contributing to delayed diagnoses, longer waiting times, and poorer health outcomes [2]. Identifying factors associated with non-attendance is therefore important for improving service delivery and patient outcomes.

Health inequalities within the NHS remain substantial, with life expectancy differing by 9.4 years in men and 7.7 years in women between the least and most deprived 10% of the population [3]. Disparities in diabetes care are also evident. Despite having higher rates of type 2 diabetes, Black and South Asian individuals are less likely to receive recommended diabetes care processes and screening [4]. These disparities suggest that demographic and structural factors may influence engagement with healthcare services. To our knowledge, no prior study has examined non-attendance determinants including ethnicity, appointment timing and geographic access, specifically within a diabetes/endocrinology population across a single NHS Trust based in North West London.

Harrow and Ealing have a higher prevalence of diabetes (9.99% and 8.95% respectively) compared with the national average of 7.1% in 2020-2021 [5]. Understanding factors associated with clinic non-attendance within these populations is therefore important to support equitable access to specialist services. This study aimed to identify demographic and service-level factors associated with outpatient non-attendance across two NHS hospitals, to inform future service improvement strategies.

## Materials and methods

Study design and setting 

This retrospective observational study was conducted within the diabetes and endocrinology outpatient services at Northwick Park Hospital and Ealing Hospital, which are part of the London North West University Healthcare NHS Trust. The study compared patients who attended scheduled outpatient appointments with those who did not attend, with the aim of identifying demographic and service-level factors associated with non-attendance.

Study population

All patients with scheduled diabetes or endocrinology outpatient appointments during this study period were eligible for inclusion, with both new and follow-up appointments included. 

Patients were excluded if they had opted out of access to their electronic patient records or if discrepancies were identified between recorded home address and registered general practice location, suggesting potential data inaccuracies or recent relocation affecting distance calculations. 

Study period and sampling strategy 

Appointments were sampled from two distinct time periods (December 2023 - January 2024 and April - July 2024) to reduce the potential impact of seasonal variation. Both new and follow-up appointments were included, and duplicate appointments per patient were allowed, though none were present in our sample. Telephone appointments were excluded because determinants of attendance may differ substantially from in-person clinic attendance. To minimise temporal and site-related bias, equal numbers of attended and non-attended appointments were sampled in a consecutive manner from clinics of the same type, within the same time period, across both hospital sites, for both attendance groups. Within each sampling window, appointments were screened sequentially in chronological order, with every eligible appointment assessed against the inclusion criteria until the target balanced numbers were reached. This balanced sampling strategy facilitated comparative analysis between attendance groups. A formal sample size calculation was not performed due to the exploratory retrospective design.

Data sources and variables 

Data were retrospectively extracted from electronic patient records within diabetes and endocrinology outpatient services. 

The variables collected included: a) Demographic variables: age, sex and ethnicity, b) Appointment-related variables: appointment date and time of day, and c) Geographic variables: home-to-hospital, general practice (GP)-to-hospital, and GP-to-home distances.

Distance measures were calculated using postcode-derived geographic distance estimates based on driving distance. All data were anonymised before analysis. The primary outcome measured was binary (attended vs non-attended).

Data extraction process 

Data extraction was performed using a standardised extraction template to promote consistency and minimise transcription errors. A subset of records was independently reviewed to ensure accuracy and consistency of data extraction. 

All variables included in the analysis were complete for the study cohort, as data were derived from routinely collected electronic health records with mandatory fields. No missing data were identified; therefore, no imputation was required. 

Variable categorisation 

Age was analysed as both a continuous and categorical variable (> 50 and ≤ 50) to facilitate subgroup comparison. Ethnicity was grouped into standard NHS categories (White, Black, Asian, Mixed, Other). 

Appointment time was dichotomised into morning (AM) and afternoon (PM) to reflect operational scheduling differences and assess potential service-level effects. 

Geographical distance was assessed using three distinct metrics: home-to-hospital, GP-to-hospital, and GP-to-home distances. Distance was categorised (<2 km, 2-5 km, 5-10 km, >10 km) for descriptive purposes only, while continuous values were used in regression analyses to preserve statistical power and avoid arbitrary threshold effects.

Outcome measures

The primary outcome of interest was outpatient non-attendance. Non-attendance was defined as failure to attend a scheduled appointment without prior cancellation or same-day rescheduling recorded in the electronic health record. Secondary analysis explored associations between non-attendance and demographic factors, appointment timing, and geographical distance measures. 

Statistical analysis

Categorical variables were compared using Fisher’s exact test, while continuous variables were analysed using the Wilcoxon rank-sum test due to non-normal distribution.

Regression model specification 

Multivariable logistic regression was performed to assess the association between distance measures (home-to-hospital, GP-to-hospital, GP-to-home) and non-attendance. Distance variables were modelled as continuous predictors. Covariates (age, sex and appointment time) were selected based on known associations with outpatient attendance and were limited to reduce the risk of model overfitting given the sample size. Due to collinearity between spatial measures, each distance variable was analysed in separate regression models, with identical covariate adjustment. The outcome was binary (attendance vs non-attendance). 

Univariate logistic regression was performed to estimate odds ratios and 95% confidence intervals for categorical variables of interest. White ethnicity and afternoon appointments were used as reference categories in regression analyses. 

Model fit was assessed using likelihood ratio testing, and results are presented as odds ratios (ORs) with 95% confidence intervals (CIs). Statistical significance was defined as p < 0.05.

Ethical considerations

This study was conducted as a retrospective analysis of anonymised routinely collected clinical data and was classified as service evaluation under local NHS governance frameworks. In accordance with institutional policy, formal research ethics committee approval was not required. No identifiable patient information was accessed or used, and all data handling complied with relevant data protection and governance standards.

## Results

Baseline characteristics 

A total of 159 outpatient appointments were analysed, comprising 80 attendees and 79 non-attendees. Of these, 80 appointments were from Northwick Park and 79 from Ealing Hospital. The cohort included 75 males (47.2%) and 84 females (52.8%), with a similar sex distribution across sites (Northwick Park: 45% male; Ealing Hospital: 44.3% male). Seventy-eight patients (49.1%) were above the age of 50. Median age was slightly higher amongst attendees compared with non-attendees. There was no evidence of age- or sex- based differences in attendance patterns in this cohort (Table 1).

**Table 1:** Baseline characteristics of participants Data are presented as median (IQR) or n (%). Continuous variables were compared using Wilcoxon rank-sum test and categorical variables using Fisher’s exact test.

Characteristics	Total (n = 159)	Attended (n = 80)	Not-Attended (n = 79)	p-value
Age, median (IQR)	48 (39-71)	49.5 (35-66)	45 (36-60)	0.382
Male, n (%)	75 (47.2%)	35 (43.8%)	40 (50.6%)	0.261
Time of day, n (%)				0.14
AM	27 (17.0%)	10 (12.5%)	17 (21.5%)	
PM	132 (83.0%)	70 (87.5%)	62 (78.5%)	
Ethnicity, n (%)				0.002
White	43 (27.0%)	31 (38.8%)	12 (15.2%)	
Black	16 (10.1%)	6 (7.5%)	10 (12.7%)	
Asian	49 (30.8%)	25 (31.3%)	24 (30.4%)	
Mixed	6 (3.8%)	4 (5.0%)	2 (2.5%)	
Other	45 (28.3%)	14 (17.5%)	31 (39.2%)	

Ethnicity

Attendance status differed significantly across ethnicities in a univariate analysis (Fisher’s exact test, p = 0.002), although subgroup sizes were small for some categories, notably Mixed ethnicity (n = 6), limiting the precision of individual group estimates. In this sampled cohort, patients identifying as Black (OR: 4.31, 95% CI: 1.28 - 14.46) and Other (OR: 5.72, 95% CI: 2.28 - 14.3) had higher odds of non-attendance compared with White patients. 72.1% of White patients attended appointments, compared with 37.5% and 31.1% of patients identifying as Black and Other ethnicity, respectively (Figure 1). 

**Figure 1:**
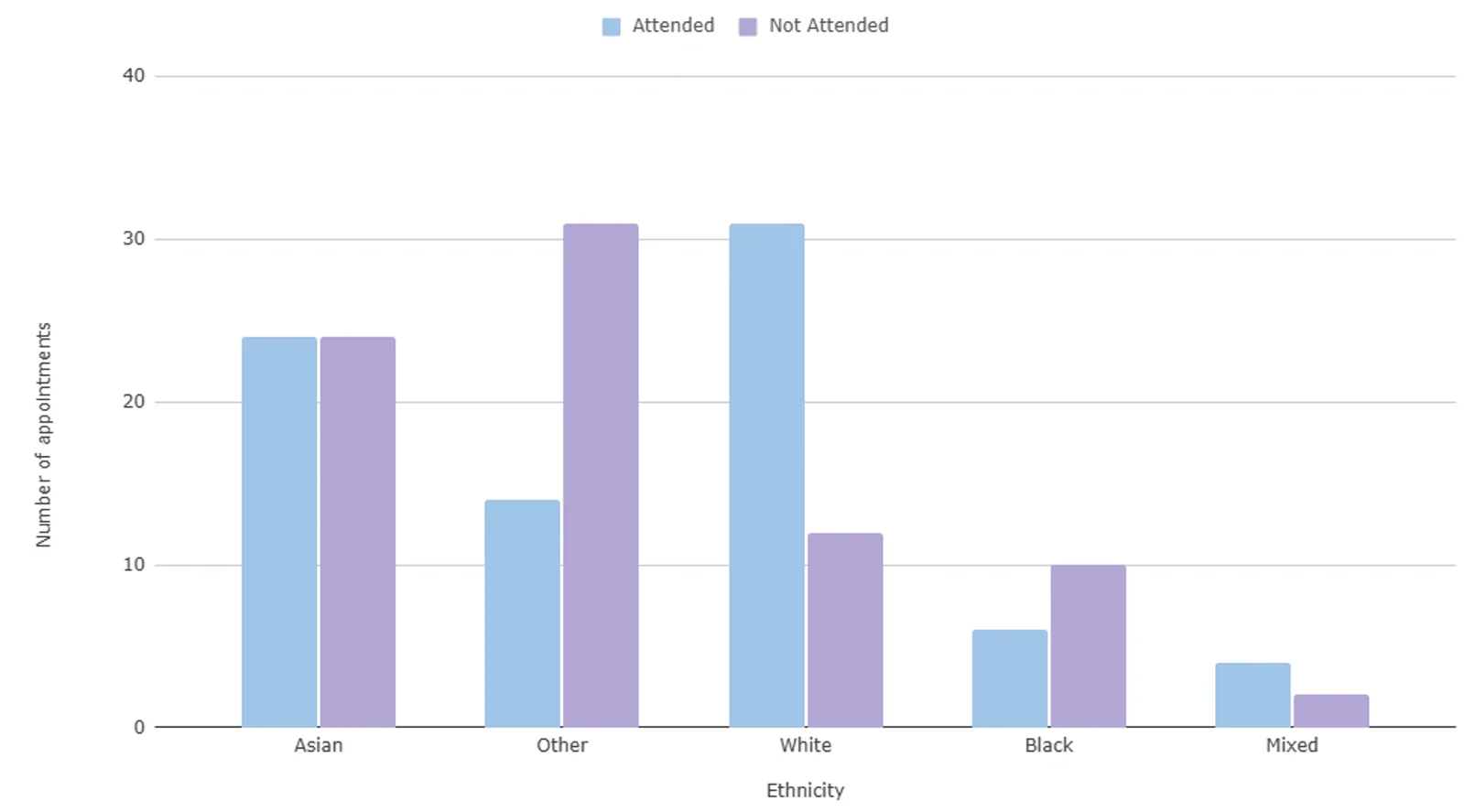
Proportion of patients by ethnicity who attended versus did not attend outpatient clinic appointments

Appointment timings 

Morning appointments were associated with higher odds of non-attendance in univariate logistic regression (OR 1.92, 95% CI 0.81- 4.50); however, this was not statistically significant (p = 0.14).

Travel proximity metrics 

No significant association was observed between any geographical distance measure and attendance status in either univariate analysis or multivariable logistic regression models (Table 2). Odds ratios were close to 1 across all models, and confidence intervals included the null value, suggesting minimal effect of travel distance on outpatient attendance within this cohort. 

**Table 2:** Multivariate logistic regression analysis of geographical distance measures and outpatient non-attendance Models were adjusted for age, sex and appointment time.

Variable	OR	95% CI	p value
Home to Clinic	1	0.99 - 1.02	0.96
Home to GP	0.94	0.82 - 1.07	0.33
GP to Clinic	1	0.99 - 1.02	0.73

Summary of findings 

Outpatient non-attendance varied significantly by ethnicity in univariate analysis (p = 0.002). Patients identifying as Black or Other ethnicity demonstrated higher odds of non-attendance compared to White patients. No evidence of association was observed between attendance status and age, sex, or geographical distance measures. Morning appointments were associated with higher odds of non-attendance, although this did not reach statistical significance.

## Discussion

Ethnicity was associated with outpatient non-attendance in univariate analysis, with higher odds of non-attendance observed among Black or Other ethnic groups. Wide confidence intervals and small subgroup sizes limit the precision of these estimates, but the direction of association is consistent with existing literature demonstrating disparities in engagement across outpatient care and hospital admissions [6-8]. 

These patterns may reflect a combination of structural and individual-level factors, though none were directly measured in this dataset. Qualitative literature points to possible contributors such as difficulty navigating appointment systems [9], ineffective communication and unreliable reminder processes [10], variation in health literacy, trust in healthcare systems, and prior experiences of care [11-12]. As ethnicity was only assessed through univariate analysis without adjustment for socioeconomic status, employment, education, language proficiency, health literacy, comorbidity burden, or prior attendance history, these explanations remain speculative, and the reported odds ratios should not be interpreted as independent effects of ethnicity. 

Morning appointments were associated with higher odds of non-attendance; however, this did not reach statistical significance and should be interpreted cautiously given the small number of morning appointments (n = 27). This null finding is consistent with previous studies reporting no reliable association between appointment timing and attendance [13] and may reflect differences in scheduling flexibility, particularly for those with work or caregiving responsibilities [13-14]. Appointment timing may therefore function as a proxy for broader accessibility constraints rather than an independent determinant of attendance. 

No association was observed between geographical distance and non-attendance across any measure examined, with odds ratios close to one in all models. This aligns with findings from Peter et al. [15], which suggest that simple proximity measures may not capture real-world access; factors such as travel time, transport availability, cost, and journey complexity are likely to be more relevant, particularly in urban settings where geographical proximity varies less and transport infrastructure may offset the impact of distance.

Together, these findings are consistent with a multifactorial model of non-attendance, in which socio-demographic and system-level factors appear more influential than geographical proximity alone. This study extends the existing literature by examining these patterns across two NHS hospital sites within a high-prevalence diabetes population, reinforcing evidence of ethnic variation in attendance and suggesting that distance-based proxies for access may have limited explanatory value in urban outpatient settings.

These findings have implications for service design. Interventions targeting non-attendance may benefit from focusing on modifiable system-level factors: flexible booking systems [10,16], enhanced reminder systems [17-19], and predictive modelling to identify at-risk patients [20] have each shown promise. Incorporating patient input into service design may be particularly valuable for groups at higher risk of non-attendance.

Several limitations should be considered. The retrospective design and reliance on routinely collected data limit the ability to account for unmeasured confounders discussed above and introduce potential misclassification, particularly in distinguishing non-attendance from late cancellations.

Ethnicity findings in particular are based on unadjusted univariate analysis; no multivariable model was fitted due to small subgroup sample sizes (Mixed, n = 6), which would have produced unstable estimates. Balanced sampling of attended and non-attended appointments means the findings cannot be used to estimate population-level non-attendance prevalence and may itself introduce selection bias affecting the observed associations. 

The absence of a formal power calculation means the study may be underpowered to detect clinically meaningful associations, particularly within smaller ethnic subgroups. Exclusion of patients with address discrepancies may have systematically excluded more transient or vulnerable populations, potentially biasing both the ethnicity and distance findings. Finally, data were derived from two hospital sites within a single NHS Trust, which may limit generalisability to other settings.

## Conclusions

This retrospective observational study examined factors associated with non-attendance in diabetes and endocrinology outpatient clinics. Attendance varied significantly by ethnicity in unadjusted univariate analysis (p = 0.002); however, given the small subgroup sizes, wide confidence intervals, and absence of adjustment for socioeconomic or other confounders, this should be regarded as hypothesis-generating rather than confirmatory evidence of an independent association between ethnicity and attendance. Morning appointments were associated with higher odds of non-attendance (OR 1.92, 95% CI 0.81-4.50), though this did not reach statistical significance and the role of appointment timing remains inconclusive. Together, these findings point to demographic variation in attendance that merits further, adjusted investigation. Future work should incorporate multivariable adjustment for socioeconomic status, employment, language proficiency and health literacy, alongside multi-centre data and qualitative insights from patient experience, to clarify the drivers of non-attendance and inform targeted intervention.
